# Divergences in Macrophage Activation Markers Soluble CD163 and Mannose Receptor in Patients With Non-cirrhotic and Cirrhotic Portal Hypertension

**DOI:** 10.3389/fphys.2021.649668

**Published:** 2021-06-11

**Authors:** Nikolaj Worm Ørntoft, Michel Blé, Anna Baiges, Jose Ferrusquia, Virginia Hernández-Gea, Fanny Turon, Marta Magaz, Søren Møller, Holger Jon Møller, Juan Carlos Garcia-Pagan, Henning Gronbaek

**Affiliations:** ^1^Department of Hepatology and Gastroenterology, European Reference Network on Hepatological Diseases (ERN RARE-LIVER), Aarhus University Hospital, Aarhus, Denmark; ^2^Barcelona Hepatic Hemodynamic Laboratory, Liver Unit, Hospital Clínic, Centro de Investigación Biomédica en Red Enfermedades Hepáticas y Digestivas (CIBEREHD), European Reference Network on Hepatological Diseases (ERN RARE-LIVER), Institut de Investigacions Biomèdiques August Pi i Sunyer (IDIBAPS), University of Barcelona, Barcelona, Spain; ^3^Center of Functional and Diagnostic Imaging and Research, Department of Clinical Physiology and Nuclear Medicine 260, Copenhagen University Hospital Hvidovre, Hvidovre, Denmark; ^4^Department of Clinical Biochemistry, Aarhus University Hospital, Aarhus, Denmark

**Keywords:** portal hypertension, cirrhosis, macrophages, non-cirrhotic portal hypertension, biomarker

## Abstract

**Introduction:**

Macrophages are involved in development and progression of chronic liver disease and portal hypertension. The macrophage activation markers soluble (s)CD163 and soluble mannose receptor (sMR), are associated with portal hypertension in patient with liver cirrhosis but never investigated in patients with non-cirrhotic portal hypertension. We hypothesized higher levels in cirrhotic patients with portal hypertension than patients with non-cirrhotic portal hypertension. We investigated sCD163 and sMR levels in patients with portal hypertension due to idiopathic portal hypertension (IPH) and portal vein thrombosis (PVT) in patients *with* and *without* cirrhosis.

**Methods:**

We studied plasma sCD163 and sMR levels in patients with IPH (*n* = 26), non-cirrhotic PVT (*n* = 20), patients with cirrhosis *without* PVT (*n* = 31) and *with* PVT (*n* = 17), and healthy controls (*n* = 15).

**Results:**

Median sCD163 concentration was 1.51 (95% CI: 1.24–1.83) mg/L in healthy controls, 1.96 (95% CI: 1.49–2.56) in patients with non-cirrhotic PVT and 2.16 (95% CI: 1.75–2.66) in patients with IPH. There was no difference between non-cirrhotic PVT patients and healthy controls, whereas IPH patients had significantly higher levels than controls (*P* < 0.05). The median sCD163 was significantly higher in the cirrhotic groups compared to the other groups, with a median sCD163 of 6.31 (95% CI: 5.16–7.73) in cirrhotics *without* PVT and 5.19 (95% CI: 4.18–6.46) *with* PVT (*P* < 0.01, all). Similar differences were observed for sMR.

**Conclusion:**

Soluble CD163 and sMR levels are elevated in patients with IPH and patients with cirrhosis, but normal in patients with non-cirrhotic PVT. This suggests that hepatic macrophage activation is more driven by the underlying liver disease with cirrhosis than portal hypertension.

## Introduction

Liver macrophages play a significant role in chronic liver disease development and progression, and are also suggested to play a role in portal hypertension (Steib, 2011). The macrophages may be activated by the specific liver disease (e.g., virus, alcohol, steatosis, and drugs) where damage associated molecular patterns (DAMPs) activate macrophages accompanied by inflammation, fibrosis, and finally cirrhosis. Further, patients with liver cirrhosis and portal hypertension have intestinal edema and a leaky gut wall resulting in translocation of endotoxins and gut bacteria, e.g., pathogen associated molecular patterns (PAMPs), stimulating gastrointestinal and liver macrophages (Wiest and Garcia-Tsao, 2005; Wiest et al., 2014; Seitz et al., 2018) with secretion of inflammatory and vasoactive cytokines (Steib et al., 2007, 2010a,b).

Similar mechanisms of macrophage activation especially PAMPs may be involved in patients with non-cirrhotic portal hypertension; however, most often, without underlying liver disease. Non-cirrhotic portal hypertension is mainly caused by vascular disorders, especially portal vein thrombosis (PVT). However, a number of other conditions are associated with non-cirrhotic portal hypertension (Wanless, 1990; Strauss and Valla, 2014; Hernandez-Gea et al., 2018), and yet in some patients, a specific cause for the portal hypertension cannot be identified and these patients are classified as having idiopathic portal hypertension (IPH) currently also known as porto sinusoidal vascular liver disease (Schouten et al., 2012b; Hernandez-Gea et al., 2018). Patients with non-cirrhotic portal hypertension may also display macrophage activation due to portal hypertension and PAMPs; however, it is unknown how macrophage activation differs between patients with non-cirrhotic portal hypertension e.g., PVT and IPH and patients with cirrhosis *with* and *without* PVT. Divergences may partly explain differences in disease severity and prognosis in patients with non-cirrhotic portal hypertension compared to patients with liver cirrhosis, who may develop acute decompensation with risk of progression toward acute-on-chronic liver failure.

As recently reviewed macrophage activation markers soluble (s)CD163 and soluble mannose receptor, sMR, are associated with chronic liver disease severity (Child-Pugh and MELD scores) and portal hypertension (Møller et al., 2016). However, the macrophage activation markers sCD163 and sMR have never been studied in the setting of non-cirrhotic portal hypertension. We therefore aimed to evaluate the role of macrophage activation by sCD163 and sMR in patients with non-cirrhotic portal hypertension (PVT and IPH) and compare this to patients with cirrhotic portal hypertension *with* and *without* PVT and in healthy controls. We hypothesized higher levels in patients with cirrhosis and portal hypertension than patients with non-cirrhotic portal hypertension, which suggest that the underlying liver disease is the main driver of macrophage activation.

## Materials and Methods

### Patients and Healthy Controls

Ninety-four patients were included in the study from 2003 to 2015 from the outpatient clinic in Barcelona. The patients were divided into four groups according to their underlying disease. Twenty-six patients had IPH, 20 patients had non-cirrhotic PVT, 31 patients had cirrhosis *without* PVT and 17 patients had cirrhosis *with* PVT. All patients with IPH reached this diagnosis after discarding other etiologies for portal hypertension with CT-examination, biochemical screening and liver biopsy. In general, the histological changes were subtle and diverse. The most pronounced histological features present in 48% of the IPH patients were portal tract vascular abnormalities (including vascular multiplication, periportal vascular channels and aberrant vessels). Hepatic sinusoidal dilation, architectural disturbance (irregular distribution of central veins and portal tract) and regenerative nodules was present in 38%, 21%, and 21%, respectively. Two patients with IPH had histological features of obliterative portal venopathy. In 21% of IPH patients there was mild perisinusoidal fibrosis. None of the patients with IPH showed histological features of inflammation in the liver biopsy. Fifteen healthy human subjects were included at Hvidovre Hospital in Denmark. Liver cirrhosis was diagnosed in patients with underlying chronic liver disease (e.g., alcohol, HCV, and HBV) combined with imaging showing nodular surface and collaterals including clinical complications to portal hypertension (e.g., ascites, varices, and hepatic encephalopathy).

All patients and healthy controls had physical examination, measurements of additional biochemical parameters and underwent hemodynamic investigation with liver vein catheterization for measurement of hepatic venous pressure gradient (HVPG), physical examination and measurements of additional biochemical parameters (Table 1). HVPG was determined as the difference between the wedged and the free hepatic venous pressure. No patients or healthy subjects had fever or other signs of infections. Blood samples were collected from a peripheral vein for measurements of sCD163 and sMR and frozen at −80°C until analysis. Informed consent was obtained from all participants according to the Helsinki Declaration.

**TABLE 1:** Baseline characteristics.

	**Controls (*n* = 15)**	**IPH (*n* = 26)**	**Non-cirrhotic PVT (*n* = 20)**	**Cirrhosis with PVT (*n* = 17)**	**Cirrhosis without PVT (*N* = 31)**
Male/female (*n*)	6/9*,#	19/7	15/5	9/8	15/16
Age (years)	53 (51, 65)	43 (31, 56)€,&	53 (38, 62)€	61 (51, 70)	60 (51, 68)
Child-Pugh score	–	5 (5, 6)€,&	5 (5, 6)€,&	7 (7, 9)	7 (5, 8)&
HVPG (mmHg)	3.0 (2.0, 4.0)*,€,&	6.8 (5.5, 11)#,€,&	4.0 (2.75, 4.5)€,&	21.5 (16, 23)	18.0 (14.5, 19.5)&
Varices (% with small and large varices)	0%; 0%*, #,€,&	12%; 77%€, &	25%; 60%€,&	12%; 76%	29%; 52%
Ascites (% with non-tense and tense ascites)	0%, 0%€,&	12%; 0%€,&	15%; 0%€,&	71%; 18%	48%; 3%&
BMI (kg/m^2^)	25 (19, 28)	23 (21, 25)€,&	23 (21, 26)€,&	25 (24, 30)	25 (23, 29)
Bilirubin (mg/dL)	0.4 (0.2, 1)€,&	1 (0.6, 1.5)	0.8 (0.6, 1.7)	1.6 (1.4, 3.0)	1.4 (0.8, 2.5)
Alkaline phosphatase (IU/l)	113 (69, 193)	142 (114, 287)	163 (135, 216)	133 (108, 286)	173 (96, 215)
Albumin (g/dl)	4.0 (3.5, 4.2)	4.0 (3.6, 4.2)€,&	4.1 (3.6, 4.4)€,&	3.6 (3.1, 3.8)	3.6 (3.2, 3.9)
INR	1.1 (1.0, 1.2)€,&	1.2 (1.1, 1.4)&	1.2 (1.1, 1.3)&	1.4 (1.3, 1.6)	1.3 (1.2, 1.4)&
AST (IU/l)	–	33 (27, 46)€	30 (25, 45)€	43 (33, 53)	83 (38, 115)&
ALT (IU/l)	28 (23, 35)€	25 (21, 42)€	29 (23, 48)€	26 (21, 38)	58 (24, 101)&
Creatinine (mg/dl)	0.84 (0.74, 1.1)	0.77 (0.61, 0.99)	0.86 (0.78, 0.94)	0.86 (0.72, 1.02)	0.70 (0.59, 0.94)
MELD score	–	9.8 (8.2, 11.7)€,&	9.5 (8.0, 10.6)€,&	13.1 (10.3, 15.2)	12.4 (10.1, 15.8)
Sodium (mmol/l)	143 (140, 145)	140 (139, 143)	140 (137, 141)€	141 (137, 142)	142 (140, 144)
Potassium (mmol/l)	4.3 (3.8, 4.4)	4.1 (3.8, 4.5)	4.4 (4.1, 4.7)	4.2 (4.0, 4.5)	4.3 (4.0, 4.6)
Hemoglobin (g/dl)	8.6 (7.8, 9.0)	12.9 (10.7, 14.5)	12.3 (10.5, 14.7)	11.2 (10.4, 12.5)	12.8 (11.3, 14.2)
Thrombocytes (10^9^/l)	372 (250, 447)*,#,€, &	82 (60, 112)#	159 (105, 281)€,&	62 (44, 102)	97 (70, 125)
Leukocytes (10^9^/l)	–	4.0 (3.4, 5.0)	5.4 (3.3, 8.4)	3.4 (2.9, 4.3)	4.0 (3.2, 5.7)

Values are reported as median with 25 and 75% interquartile range in brackets unless other is specified.

* Significantly different from patients with IPH (*P* < 0.05).

*# Significantly different from patients with non-cirrhotic PVT (*P* < 0.05).*

*€ Significantly different from patients with cirrhosis without PVT (*P* < 0.05).*

*& Significantly different from patients with cirrhosis with PVT (*P* < 0.05).*

### Soluble CD163 and Soluble MR

Levels of sCD163 and sMR in plasma samples were measured by an in-house sandwich enzyme-linked immunosorbent assay as previously described (Moller et al., 2002; Rodgaard-Hansen et al., 2014).

### Statistics

Normality was assessed visually by using quantile-quantile-plots and histograms. The values of the biomarkers were not normally distributed. To obtain normal distribution, the data were log-transformed. Accordingly, data are presented as median with a 95% CI of the median. Multiple linear regression was used to test if the values of sCD163 and sMR were different between the groups. Age was used as a control variable in the regression of both sCD163 and sMR, as sCD163 is known to increase with age (Moller, 2012). Model assumptions was checked and fulfilled. For all other parameters the Mann–Whitney *U* test was used to test statistical differences between groups. A *p*-value < 0.05 was considered to indicate statistical significance. Statistical analyses were performed using STATA software, release 11 (StataCorp, College Station, TX, United States).

## Results

### Patient Groups

Gender was evenly distributed for the healthy controls and the patients with cirrhosis, but in the groups with IPH and non-cirrhotic PVT there was an overweight of males (75%). Although HVPG was significantly higher in the patients with IPH compared to healthy controls and patients with non-cirrhotic PVT, there was no difference in the clinical signs of portal hypertension, with no significantly difference in the degree of varices or the degree of ascites between the groups (*P* = 0.26 and *P* = 0.73, Table 1). HVPG was significantly higher in the patient groups with cirrhosis compared to the non-cirrhotic patients (*P* < 0.05) and they also had a significantly higher degree of ascites (*P* < 0.05 for all), whereas there was no significantly difference in the grade of varices (*P* > 0.05, for all, Table 1). Both HVPG and the degree of ascites was significantly higher in the cirrhotic group *with* PVT compared to the cirrhotic group *without* PVT (*P* = 0.02 and *P* = 0.006, Table 1).

### Soluble CD163

**FIGURE 1:**
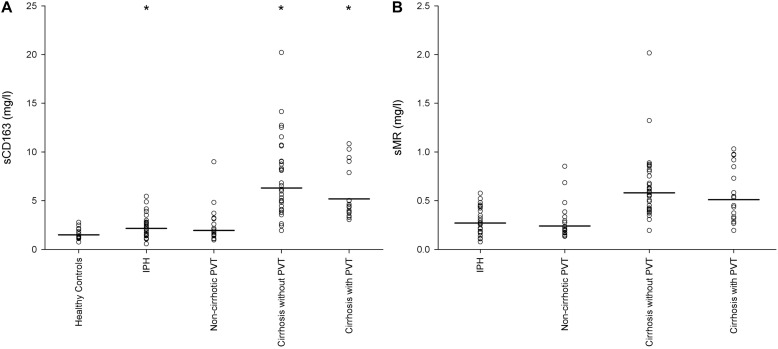
Plasma concentration and median s163 **(A)** and sMR **(B)** levels in patients with idiopathic portal hypertension (IPH), non-cirrhotic portal vein thrombosis, cirrhosis without portal vein thrombosis, cirrhosis with portal vein thrombosis and healthy controls. sCD163 was significantly elevated in IPH and the two cirrhosis groups when compared to healthy controls (*P* < 0.05). Both sCD163 and sMR was significantly higher in the cirrhosis groups compared to the non-cirrhosis groups (*P* < 0.01). There was no difference between the two cirrhosis groups. *P < 0.05 compared to healthy controls.

In the healthy controls and in patients with non-cirrhotic PVT, where the liver can be assumed to be normal or near normal, the plasma concentration of sCD163 was low and within the normal range (0.69–3.86 mg/L). The median plasma concentration was 1.51 mg/L (1.24–1.83) in healthy controls and 1.96 mg/L (1.49–2.56) in patients with non-cirrhotic PVT (Figure 1A); and with no significant difference between the two groups (*P* = 0.09).

In the patients with IPH sCD163 was slightly but significantly elevated [2.16 mg/L (1.75–2.66)], when compared to the healthy controls (*P* = 0.007) (Figure 1A). The median plasma sCD163 concentration in patients with IPH was slightly but insignificantly higher than patients with non-cirrhotic PVT (*P* = 0.35).

In patients with cirrhosis sCD163 levels were high with a median of 6.31 mg/L (5.16–7.73) in the patients *without* PVT and 5.19 mg/L (4.18–6.46) in the patients *with* PVT (Figure 1A). The two patient groups with cirrhosis had significantly elevated sCD163 compared to healthy controls, patients with IPH and patients with non-cirrhotic PVT (all *P* < 0.001). There was no difference between the concentration of sCD163 in the cirrhotic patients *with* or *without* PVT (*P* = 0.23).

### Soluble MR

Soluble mannose receptor was not measured in healthy controls, but reference values have been established with a mean of 0.28 mg/L and 95% reference interval of 0.10 mg/L to 0.43 mg/L (Rodgaard-Hansen et al., 2014). The median concentration of sMR was 0.27 mg/L (0.22–0.33) in patients with IPH and 0.24 mg/L (0.19–0.31) in the patients with non-cirrhotic PVT (Figure 1B). In both cirrhotic patient groups, median sMR was approximately two times higher than the concentration in the patients without cirrhosis with 0.58 mg/L (0.49–0.68) in cirrhotic patients *without* PVT and 0.51 mg/L (0.39–0.66) in cirrhotic patients *with* PVT (Figure 1B).

Similar, to the results of sCD163 the median concentration of sMR was higher but not significantly different in patients with IPH compared to patients with non-cirrhotic PVT (*P* = 0.46) and there was no difference between the cirrhosis groups (*P* = 0.41). The concentration of sMR was significantly higher in both patient groups with cirrhosis compared to the patients with IPH and non-cirrhotic PVT (*P* < 0.001 for all cases), see Figure 1B.

### Hepatic Venous Pressure Gradient

Median HVPG was 3.0 mmHg in the healthy controls, 6.8 mmHg in patients with IPH, and 4.0 mmHg in patients with non-cirrhotic PVT (Table 1). In the patients with cirrhosis, median HVPG was 18.0 mmHg in patients without PVT and 21.5 mmHg in patient with PVT (Table 1). There was no correlation between HVPG and sCD163. Soluble MR was only correlated to HVPG in patients with cirrhosis without PVT (Rho = 0.46, *P* = 0.01).

## Discussion

This is to our knowledge the first study to investigate macrophages and macrophage activation markers in patients with IPH and non-cirrhotic PVT. The main finding of the present study was the significantly elevated sCD163 levels in patients with underlying liver disease and cirrhosis *with* and *without* PVT, and being lesser in IPH and non-cirrhotic PVT. This may suggest that the primary driver for hepatic macrophage activation and elevated sCD163 and sMR levels is the underlying liver disease with cirrhosis rather than portal hypertension.

A major strength of the present study is the inclusion of well-characterized patients and healthy controls who all had invasive measurements of HVPG. The study limitations include the relatively small number of patients within each category and the cross-sectional design, which does not permit determination of changes in macrophage activation marker levels with prognosis or progression or regression of inflammation and fibrosis. However, this may not affect the rather clear distinction between healthy controls and patients with IPH or cirrhosis. Additionally, while included patients had stable disease it is not possible to control for subclinical events, such as minor infections, which could affect macrophage activation; however, none of the patients showed any signs of infections at inclusion or during HVPG measurements.

CD163 is a monocyte/macrophage lineage specific scavenger receptor for the hemoglobin and haptoglobin complex (Kristiansen et al., 2001). The soluble form is present in plasma under normal circumstances but substantially increased during macrophage activation (Moller, 2012). Over the past decade, studies have established macrophage activation as an important factor in liver disease development, progression and prognosis. Macrophage activation, as measured by sCD163, is associated with liver fibrosis and cirrhosis, liver disease severity (Child-Pugh- and MELD scores), and portal hypertension (Holland-Fischer et al., 2011; Gronbaek et al., 2012; Rode et al., 2013; Kazankov et al., 2014, 2015, 2016; Grønbæk et al., 2020). Furthermore, sCD163 levels are associated with prognosis, predict the risk of variceal bleeding (Rode et al., 2013; Waidmann et al., 2013) and correlates to disease severity and treatment response in patients with non-alcoholic fatty liver disease (Kazankov et al., 2015), alcoholic hepatitis (Sandahl et al., 2014), hepatitis B and C virus infection (Dultz et al., 2015; Laursen et al., 2018, 2019) and autoimmune liver diseases (Gronbaek et al., 2016a; Bossen et al., 2020, 2021). The magnitude of macrophage activation and corresponding elevated plasma markers depends on the underlying pathogenesis, being more pronounced in conditions with a high hepatic inflammatory load and fibrosis like acute liver failure and advanced cirrhosis (Hiraoka et al., 2005; Moller et al., 2007; Gronbaek et al., 2012). In acute liver failure, macrophage activation is dynamic and resolves with disease regression in contrast to the stable increase in activation seen in advanced cirrhosis (Hiraoka et al., 2005).

The mannose receptor is able to bind various ligands of microbial and endogenous origin and is involved in antigen presentation and macrophage activation (Martinez-Pomares, 2012). The receptor is expressed on selected inflammatory cells, including subsets of macrophages, dendritic and endothelial cells and shed during inflammation and subsequently measurable as soluble MR (Martinez-Pomares, 2012; Rodgaard-Hansen et al., 2014). Consequently, sMR is not as specific a marker of macrophage activation as sCD163. However, elevated sMR levels have previously been described in patients with liver disease and are shown to be associated to disease severity, portal hypertension and mortality (Gronbaek et al., 2016b; Laursen et al., 2017; Sandahl et al., 2017; Grønbaek et al., 2021).

Hepatic venous pressure gradient measures the post-sinusoidal pressure gradient. Consequently, the patients without cirrhosis who have elevated pre-sinusoidal pressure and a normal pressure gradient across the liver, e.g., patients with non-cirrhotic PVT or IPH, can still suffer from significant splenic and portal hypertension without it being detectable with standard HVPG-measurement (Keiding et al., 2004; Sørensen et al., 2018). The patients with IPH had a significantly higher HVPG compared to healthy controls (Table 1), but no association to macrophage activation, as measured by sCD163 and sMR. Same lack of association was seen for cirrhotic patients, except for cirrhotic patients without PVT where HVPG correlated with sMR. This is in opposition to previous findings in patients with liver cirrhosis (Holland-Fischer et al., 2011; Gronbaek et al., 2012) and could pertain to the small sample size.

As previously observed, macrophage activation was substantially higher in patients with underlying chronic liver disease and cirrhosis compared to controls and as a novel finding, this also applied to patients with IPH where the structural changes, however, are less severe. This may suggest that macrophage activation is a pronounced feature of the underlying liver disease *per se* and not related to vascular or hemodynamic changes. In the cirrhotic patients, there was a tendency toward less macrophage activation in the group with PVT, who had a significantly higher HVPG and Child-Pugh score. This likewise supports that vascular changes are less important for macrophage activation. Additionally, findings of comparable patterns for both sCD163 and sMR corroborate the observed associations. Furthermore, we suggest that the central mechanisms behind macrophage activation in cirrhosis and IPH is not only driven by translocation of gut-derived PAMPs but represents a constitutive inflammatory upregulation in the liver disease as also demonstrated in TIPS treated patients (Holland-Fischer et al., 2011). In addition to the constitutive macrophage activation, this may be further enhanced by a general systemic inflammatory state as seen in cirrhosis, leading to immune activation and production of pro-inflammatory cytokines, like tumor necrosis factor and interleukin 8, which are known to be involved in recruitment of inflammatory cells to the liver (Seitz et al., 2018). In the event of acute exacerbation of inflammation or infection as seen in e.g., ACLF we have observed even more pronounced macrophage activation by sCD163 and sMR levels (Gronbaek et al., 2016b).

Patients with IPH in general have a better prognosis than patients with cirrhosis with 10-year transplant free survival of 82% (Siramolpiwat et al., 2014). The current treatment of IPH is restricted to the management of portal hypertension and this does not prevent disease progression (Schouten et al., 2012a; Hernandez-Gea et al., 2018). Our study shows that macrophages to some degree are activated in IPH patients and consequently therapeutic strategies aimed at decreasing macrophage activation might be relevant in the treatment of IPH in the future. However, the association between IPH and macrophage activation needs further investigations.

## Conclusion

Macrophage activation, as measured by elevated sCD163 and sMR, were only observed in patients with cirrhosis with and without PVT and in IPH patients, and not in patients with non-cirrhotic PVT. This suggest that the main determinant of macrophage activation in chronic inflammatory liver diseases is associated to the underlying liver disease with cirrhosis and not portal hypertension.

## Data Availability Statement

The original contributions presented in the study are included in the article/supplementary material, further inquiries can be directed to the corresponding author/s.

## Ethics Statement

The studies involving human participants were reviewed and approved by the Institutional Review Board, Hospital Clinic, Barcelona. The Informed consent was obtained from all participants according to the Helsinki Declaration. The patients/participants provided their written informed consent to participate in this study.

## Author Contributions

NØ: data analysis and drafting the manuscript. MB, AB, JF, VH-G, FT, MM, SM, and HM: patient collection and data aquisition. JG-P and HG: concept and design, data analysis, and finalising the manuscript. All authors approved the final version of the manuscript.

## Conflict of Interest

The authors declare that the research was conducted in the absence of any commercial or financial relationships that could be construed as a potential conflict of interest.

## Abbreviations

IPH: idiopathic portal hypertension
PVT: portal vein thrombosis
PAMPs: pathogen associated molecular patterns
TLR: toll like receptors
sCD163: soluble CD163
sMR: soluble mannose receptor
HVPG: hepatic venous pressure gradient.

## References

[B1] Bossen L. Rebora P. Bernuzzi F. Jepsen P. Gerussi A. Andreone P. (2020). Soluble CD163 and mannose receptor as markers of liver disease severity and prognosis in patients with primary biliary cholangitis. *Liver Int.* 40 1408–1414. 10.1111/liv.14466 32279422

[B2] Bossen L. Vesterhus M. Hov J. R. Färkkilä M. Rosenberg W. M. Møller H. J. (2021). Circulating Macrophage Activation Markers Predict Transplant-Free Survival in Patients With Primary Sclerosing Cholangitis. *Clin. Transl. Gastroenterol.* 12:e00315. 10.14309/ctg.0000000000000315 33646203 PMC7925135

[B3] Dultz G. Gerber L. Farnik H. Berger A. Vermehren J. Pleli T. (2015). Soluble CD163 is an indicator of liver inflammation and fibrosis in patients chronically infected with the hepatitis B virus. *J Viral. Hepat.* 22 427–432. 10.1111/jvh.12309 25181653

[B4] Grønbæk H. Gantzel R. H. Laursen T. L. Kazankov K. Møller H. J. (2020). Macrophage markers and innate immunity in cirrhosis. *J. Hepatol.* 73 1586–1588. 10.1016/j.jhep.2020.07.033 32994078

[B5] Gronbaek H. Kreutzfeldt M. Kazankov K. Jessen N. Sandahl T. Hamilton-Dutoit S. (2016a). Single-centre experience of the macrophage activation marker soluble (s)CD163 - associations with disease activity and treatment response in patients with autoimmune hepatitis. *Aliment Pharmacol. Ther.* 44 1062–1070. 10.1111/apt.13801 27679428

[B6] Grønbaek H. Møller H. J. Saliba F. Zeuzem S. Albillos A. Ariza X. (2021). Improved prediction of mortality by combinations of inflammatory markers and standard clinical scores in patients with acute-on-chronic liver failure and acute decompensation. *J. Gastroenterol. Hepatol.* 36 240–248. 10.1111/jgh.15125 32478437

[B7] Gronbaek H. Rodgaard-Hansen S. Aagaard N. K. Arroyo V. Moestrup S. K. Garcia E. (2016b). Macrophage activation markers predict mortality in patients with liver cirrhosis without or with acute-on-chronic liver failure (ACLF). *J. Hepatol.* 64 813–822. 10.1016/j.jhep.2015.11.021 26639396

[B8] Gronbaek H. Sandahl T. D. Mortensen C. Vilstrup H. Moller H. J. Moller S. (2012). Soluble CD163, a marker of Kupffer cell activation, is related to portal hypertension in patients with liver cirrhosis. *Aliment Pharmacol. Ther.* 36 173–180. 10.1111/j.1365-2036.2012.05134.x 22591184

[B9] Hernandez-Gea V. Baiges A. Turon F. Garcia-Pagan J. C. (2018). Idiopathic Portal Hypertension. *Hepatology* 68 2413–2423. 30066417 10.1002/hep.30132

[B10] Hiraoka A. Horiike N. Akbar S. M. Michitaka K. Matsuyama T. Onji M. (2005). Soluble CD163 in patients with liver diseases: very high levels of soluble CD163 in patients with fulminant hepatic failure. *J. Gastroenterol.* 40 52–56. 10.1007/s00535-004-1493-8 15692789

[B11] Holland-Fischer P. Gronbaek H. Sandahl T. D. Moestrup S. K. Riggio O. Ridola L. (2011). Kupffer cells are activated in cirrhotic portal hypertension and not normalised by TIPS. *Gut* 60 1389–1393. 10.1136/gut.2010.234542 21572121

[B12] Kazankov K. Barrera F. Møller H. J. Bibby B. M. Vilstrup H. George J. (2014). Soluble CD163, a macrophage activation marker, is independently associated with fibrosis in patients with chronic viral hepatitis B and C. *Hepatology* 60 521–530. 10.1002/hep.27129 24623375

[B13] Kazankov K. Barrera F. Moller H. J. Rosso C. Bugianesi E. David E. (2016). The macrophage activation marker sCD163 is associated with morphological disease stages in patients with non-alcoholic fatty liver disease. *Liver. Int.* 36 1549–1557. 10.1111/liv.13150 27102725

[B14] Kazankov K. Tordjman J. Moller H. J. Vilstrup H. Poitou C. Bedossa P. (2015). Macrophage activation marker soluble CD163 and non-alcoholic fatty liver disease in morbidly obese patients undergoing bariatric surgery. *J. Gastroenterol. Hepatol.* 30 1293–1300. 10.1111/jgh.12943 25772748

[B15] Keiding S. Solvig J. Gronbaek H. Vilstrup H. (2004). Combined liver vein and spleen pulp pressure measurements in patients with portal or splenic vein thrombosis. *Scand. J. Gastroenterol.* 39 594–599. 10.1080/00365520410005171 15223686

[B16] Kristiansen M. Graversen J. H. Jacobsen C. Sonne O. Hoffman H. J. Law S. K. (2001). Identification of the haemoglobin scavenger receptor. *Nature* 409 198–201. 10.1038/35051594 11196644

[B17] Laursen T. L. Rodgaard-Hansen S. Moller H. J. Mortensen C. Karlsen S. Nielsen D. T. (2017). The soluble mannose receptor is released from the liver in cirrhotic patients, but is not associated with bacterial translocation. *Liver Int.* 37 569–575. 10.1111/liv.13262 27706896

[B18] Laursen T. L. Siggaard C. B. Kazankov K. Sandahl T. D. Moller H. J. Tarp B. (2019). Time-dependent improvement of liver inflammation, fibrosis and metabolic liver function after successful direct-acting antiviral therapy of chronic hepatitis C. *J. Viral. Hepat.* 27 28–35. 10.1111/jvh.13204 31502741

[B19] Laursen T. L. Wong G. L. Kazankov K. Sandahl T. Moller H. J. Hamilton-Dutoit S. (2018). Soluble CD163 and mannose receptor associate with chronic hepatitis B activity and fibrosis and decline with treatment. *J. Gastroenterol. Hepatol.* 33 484–491. 10.1111/jgh.13849 28618015

[B20] Martinez-Pomares L. (2012). The mannose receptor. *J. Leukoc. Biol.* 92 1177–1186. 22966131 10.1189/jlb.0512231

[B21] Moller H. J. (2012). Soluble CD163. *Scand. J. Clin. Lab. Invest.* 72 1–13. 22060747 10.3109/00365513.2011.626868

[B22] Moller H. J. Gronbaek H. Schiodt F. V. Holland-Fischer P. Schilsky M. Munoz S. (2007). Soluble CD163 from activated macrophages predicts mortality in acute liver failure. *J. Hepatol.* 47 671–676. 10.1016/j.jhep.2007.05.014 17629586 PMC2179895

[B23] Moller H. J. Hald K. Moestrup S. K. (2002). Characterization of an enzyme-linked immunosorbent assay for soluble CD163. *Scand. J. Clin. Lab. Invest.* 62 293–299. 10.1080/003655102760145852 12476928

[B24] Møller H. J. Kazankov K. Rødgaard-Hansen S. Nielsen M. C. Sandahl T. Vilstrup H. (2016). “Soluble CD163 (sCD163): Biomarker of Kupffer Cell Activation in Liver Disease,” in *Biomarkers in liver disease*, ed. Preedy V. R. (Dordrecht: Springer Science+Business Media).

[B25] Rode A. Nicoll A. Moller H. J. Lim L. Angus P. W. Kronborg I. (2013). Hepatic macrophage activation predicts clinical decompensation in chronic liver disease. *Gut* 62 1231–1232. 10.1136/gutjnl-2012-304135 23442440

[B26] Rodgaard-Hansen S. Rafique A. Christensen P. A. Maniecki M. B. Sandahl T. D. Nexo E. (2014). soluble form of the macrophage-related mannose receptor (MR/CD206) is present in human serum and elevated in critical illness. *Clin. Chem. Lab. Med.* 52 453–461. 24114918 10.1515/cclm-2013-0451

[B27] Sandahl T. D. Gronbaek H. Moller H. J. Stoy S. Thomsen K. L. Dige A. K. (2014). Hepatic macrophage activation and the LPS pathway in patients with alcoholic hepatitis: a prospective cohort study. *Am. J. Gastroenterol.* 109 1749–1756. 10.1038/ajg.2014.262 25155228

[B28] Sandahl T. D. Stoy S. H. Laursen T. L. Rodgaard-Hansen S. Moller H. J. Moller S. (2017). The soluble mannose receptor (sMR) is elevated in alcoholic liver disease and associated with disease severity, portal hypertension, and mortality in cirrhosis patients. *PLoS One* 12:e0189345. 10.1371/journal.pone.0189345 29236785 PMC5728513

[B29] Schouten J. N. Nevens F. Hansen B. Laleman W. van den Born M. Komuta M. (2012a). Idiopathic noncirrhotic portal hypertension is associated with poor survival: results of a long-term cohort study. *Aliment Pharmacol. Ther.* 35 1424–1433. 10.1111/j.1365-2036.2012.05112.x 22536808

[B30] Schouten J. N. Van der Ende M. E. Koeter T. Rossing H. H. Komuta M. Verheij J. (2012b). Risk factors and outcome of HIV-associated idiopathic noncirrhotic portal hypertension. *Aliment Pharmacol. Ther.* 36 875–885. 10.1111/apt.12049 22971050

[B31] Seitz H. K. Bataller R. Cortez-Pinto H. Gao B. Gual A. Lackner C. (2018). Alcoholic liver disease. *Nat. Rev. Dis. Primers* 4:16. 10.1038/s41572-018-0014-7 30115921

[B32] Siramolpiwat S. Seijo S. Miquel R. Berzigotti A. Garcia-Criado A. Darnell A. (2014). Idiopathic portal hypertension: natural history and long-term outcome. *Hepatology* 59 2276–2285. 10.1002/hep.26904 24155091

[B33] Sørensen M. Larsen L. P. Villadsen G. E. Aagaard N. K. Grønbæk H. Keiding S. (2018). β-Blockers Improve Presinusoidal Portal Hypertension. *Dig. Dis. Sci.* 63 3153–3157. 10.1007/s10620-018-5186-1 30003386 PMC6182445

[B34] Steib C. J. (2011). Kupffer cell activation and portal hypertension. *Gut* 60 1307–1308. 10.1136/gut.2011.242560 21708827

[B35] Steib C. J. Bilzer M. Hartl J. M. Beitinger F. Gulberg V. Goke B. (2010a). Kupffer cell activation by hydrogen peroxide: a new mechanism of portal pressure increase. *Shock* 33 412–418. 10.1097/shk.0b013e3181b85934 20118678

[B36] Steib C. J. Gerbes A. L. Bystron M. Op den Winkel M. Hartl J. Roggel F. (2007). Kupffer cell activation in normal and fibrotic livers increases portal pressure via thromboxane A(2). *J. Hepatol.* 47 228–238. 10.1016/j.jhep.2007.03.019 17573142

[B37] Steib C. J. Hartmann A. C. Hesler C. Benesic A. Hennenberg M. Bilzer M. (2010b). Gerbes AL. Intraperitoneal LPS amplifies portal hypertension in rat liver fibrosis. *Lab. Invest.* 90 1024–1032. 10.1038/labinvest.2010.60 20212458

[B38] Strauss E. Valla D. (2014). Non-cirrhotic portal hypertension–concept, diagnosis and clinical management. *Clin. Res. Hepatol. Gastroenterol.* 38 564–569. 10.1016/j.clinre.2013.12.012 24581591

[B39] Waidmann O. Brunner F. Herrmann E. Zeuzem S. Piiper A. Kronenberger B. (2013). Macrophage activation is a prognostic parameter for variceal bleeding and overall survival in patients with liver cirrhosis. *J. Hepatol.* 58 956–961. 10.1016/j.jhep.2013.01.005 23333526

[B40] Wanless I. R. (1990). Micronodular transformation (nodular regenerative hyperplasia) of the liver: a report of 64 cases among 2,500 autopsies and a new classification of benign hepatocellular nodules. *Hepatology* 11 787–797. 10.1002/hep.1840110512 2189821

[B41] Wiest R. Garcia-Tsao G. (2005). Bacterial translocation (BT) in cirrhosis. *Hepatology* 41 422–433. 10.1002/hep.20632 15723320

[B42] Wiest R. Lawson M. Geuking M. (2014). Pathological bacterial translocation in liver cirrhosis. *J. Hepatol.* 60 197–209. 10.1016/j.jhep.2013.07.044 23993913

